# Draft Genome Sequence of *Zhihengliuella* sp. Strain ISTPL4, a Psychrotolerant and Halotolerant Bacterium Isolated from Pangong Lake, India

**DOI:** 10.1128/genomeA.01533-17

**Published:** 2018-02-01

**Authors:** Arti Mishra, Gopaljee Jha, Indu Shekhar Thakur

**Affiliations:** a School of Environmental Sciences, Jawaharlal Nehru University, New Delhi, India; b National Institute of Plant Genome Research, Aruna Asaf Ali Marg, New Delhi, India

## Abstract

*Zhihengliuella* sp. strain ISTPL4, a psychrotolerant bacterium, was isolated from brackish water of the high-altitude Pangong Lake in India. In this study, we report its draft genome sequence, which contains 3,529,629 bp with a G+C content of 69.84%. The genome is enriched in genes associated with cold adaptation and plant growth promotion.

## GENOME ANNOUNCEMENT

The genus *Zhihengliuella* belongs to the family *Micrococcaceae* of the class *Actinobacteria*. To the best of our knowledge, only six species have been reported to date, and they were isolated from various habitats, suggesting wide distribution of this genus (1). *Zhihengliuella* sp. strain ISTPL4 was isolated from Pangong Lake, India, which is situated at a high altitude (4,350 m) and represents diversified environments at low temperatures. It is therefore a rich source of psychrophiles. The high altitude offers an oxygen-deficient environment; however, the lake has a terrain from which collecting samples is very difficult, so few studies of such habitats have been performed. Phylogenetic analysis based on 16S rRNA gene sequencing revealed that strain ISTPL4 belongs to the genus *Zhihengliuella*. This strain is a Gram-positive rod-shaped pale-yellow-pigmented bacterium. A few strains of *Zhihengliuella* have been reported to enhance plant growth under saline stress through reduction of ethylene production via 1-aminocyclopropane-1-carboxylate deaminase activity (2, 3). Here, we report the whole-genome sequence of *Zhihengliuella* sp. ISTPL4, which, to the best of our knowledge, provides the first genome sequence information for the genus *Zhihengliuella*.

The draft genome of *Zhihengliuella* sp. ISTPL4 was sequenced using the Illumina NextSeq 500 sequencing system with a paired-end library, generating a total of 10,134,928 paired-end reads. After quality trimming, error correction, and filtering of raw reads, we obtained 9,081,677 high-quality paired-end reads. Using SPAdes v3.10.1 and Velvet (4), genome sequence processing and assembly were performed. The primary assembled contigs were subjected to scaffolding and gap filling using Baseclear SSPACE standard (5) and Baseclear GapFiller (6), respectively, and by this process we assembled the genome into one scaffold of 3,529,629 bp, with an *N*_50_ scaffold size of 3,529,629 bp.

The total genome size of *Zhihengliuella* sp. ISTPL4 is 3,529,629 bp, as assessed by a k-mer counting tool, and coverage of 860× was achieved. The strain had a G+C content of 69.84%. Genome annotation using Prokka (7) predicted 3,363 protein-coding sequences, 50 tRNAs, 4 rRNAs, 1 transfer-messenger RNA (tmRNA), and 9 miscellaneous other RNAs. Based on the annotation, CVTree (8) was used to create a phylogenetic tree using the *Micrococcaceae* lineage, which consists of 260 genomes, including the *Zhihengliuella* sp. genome. The analysis reinforced the identification of strain ISTPL4 as a *Zhihengliuella* species.

The genome was found to be enriched in genes for glycolysis, the tricarboxylic acid cycle, the pentose phosphate pathway, carbohydrate metabolism, and amino acid biosynthesis. The genome was also predicted to have genes related to cold shock proteins (such as *cspA*), pyruvate dehydrogenase (*aceE* and *aceF*), phosphatidate cytidylyltransferase (*csdA*), ribosome-binding factor A (*rbfA*), transcription termination/antitermination protein (*nusA*), and salt tolerance (such as trehalose synthase [*treS*], glycine betaine transporter [*betP*], and the Na^+^/H^+^ antiporter [*nhaA*]). It harbors genes associated with plant growth-promoting rhizobacterium (PGPR) traits; among these, the genes related to phosphate uptake, indole-3-acetic acid (IAA) synthesis, and siderophore production were especially noteworthy. The strain ISTPL4, being psychrotolerant and halotolerant, may have diverse potential biotechnological applications. Psychrophilic enzymes, such as alpha amylase and aminopeptidase, have higher *k*_cat_ values than those of the thermophilic enzymes required for industrial applications (9). Detailed genomic analyses of this bacterium will help in identifying novel compounds and revealing their potential utilization.

### Accession number(s).

This whole-genome shotgun project has been deposited at DDBJ/EMBL/GenBank under the accession number CP025422.
